# A prospective study of chronic postsurgical pain in elderly patients: incidence, characteristics and risk factors

**DOI:** 10.1186/s12877-023-04006-w

**Published:** 2023-05-12

**Authors:** Juying Jin, Ting Zhang, Xianwei Xiong, Huan Chen, Yiling Jiang, Shuangyu He

**Affiliations:** Department of Anesthesiology, The First Affiliated Hospital of Chongqing Medical University, 1 Youyi Road, Chongqing, 400016 China

**Keywords:** Chronic postsurgical pain, Elderly patients, Incidence, Risk factor

## Abstract

**Background:**

Due to the continued growth of surgical procedures in older adults and the significant impact of chronic postsurgical pain (CPSP), it is crucial to improve our understanding of the occurrence of CPSP as well as the appropriate prevention and treatment. We therefore conducted this study to determine the incidence, characteristics and risk factors of CPSP in elderly patients at both 3 and 6 months after surgery.

**Methods:**

Elderly patients (aged ≥ 60 years) undergoing elective surgery in our institution between April 2018 and March 2020 were prospectively enrolled in this study. Data on demographics, preoperative psychological well-being, intraoperative surgical and anesthesia management, and acute postoperative pain intensity were collected. At 3 and 6 months after surgery, patients received telephone interview and completed the questionnaires regarding chronic pain characteristics, analgesic consumption, and interference of the pain with activities of daily living (ADL).

**Results:**

A total of 1065 elderly patients were followed up for 6 postoperative months and included in final analysis. At 3 and 6 months after operation, the incidence of CPSP was 35.6% [95% confidence interval (95% CI) 32.7 − 38.8%] and 21.5% (95% CI 19.0% − 23.9%), respectively. CPSP cause negative impacts on patient’s ADL and most particularly on mood. Neuropathic features were found in 45.1% of the patients with CPSP at 3 months. At 6 months, 31.0% of those with CPSP reported that the pain had neuropathic features. Preoperative anxiety [3 months: Odds ratio (OR) 2.244, 95% CI 1.693 to 2.973; 6 months: OR 2.397, 95% CI 1.745 to 3.294], preoperative depression (3 months: OR 1.709, 95% CI 1.292 to 2.261; 6 months: OR 1.565, 95% CI 1.136–2.156), orthopedic surgery (3 months: OR 1.927, 95% CI 1.112 to 3.341; 6 months: OR 2.484, 95% CI 1.220 to 5.061), higher pain severity on movement within postoperative 24 h (3 months: OR 1.317, 95% CI 1.191 to 1.457; 6 months: OR 1.317, 95% CI 1.177 to 1.475) were associated with a higher risk for CPSP independently at both 3 and 6 months after surgery.

**Conclusions:**

CPSP is a common postoperative complication in elderly surgical patients. Preoperative anxiety and depression, orthopedic surgery, and greater intensity of acute postoperative pain on movement are associated with an increased risk for CPSP. It should be kept in mind that developing psychological interventions to reduce anxiety and depression and optimizing the management of acute postoperative pain will be effective in reducing the development of CPSP in this population.

## Background

Chronic postsurgical pain (CPSP) is defined as pain lasting ≥ 3 postoperative months by International Association for the Study of Pain (IASP), which is not otherwise relevant to pre-existing problems or post-surgery complications [1]. The CPSP can occur in 10–50% of patients depending on the surgical type, with 5–10% of these patients reporting severe pain [2–5]. CPSP is increasingly recognized as a public health issue and adversely impacts the patient’s long-term outcomes, physical functioning, and quality of life [6–8]. Although the mechanism underlying CPSP remains incompletely understood, a continuous inflammatory response and/or neuronal damage occurring during surgery may alter the patient’s sense of pain [9]. Neuropathic pain syndromes are more frequent among patients developing CPSP [10, 11].

On the other hand, populations are getting older across the globe. Older adults (aged ≥ 60 years) are the fastest growing section of the population and individuals in this age group undergo surgery more frequently than those in younger age group [12]. The elderly surgical patients are particularly vulnerable to postoperative complications and CPSP is one of the significant concerns in this population [13–15].

While the incidence and characteristics of postoperative acute pain in the elderly has been widely studied, only few, and mostly retrospective studies emphasized on CPSP in elderly patients [16–20]. Furthermore, the risk factors for CPSP in this population remain unclear. Due to the continued growth of surgical procedures in older individuals and the significant impact of CPSP, there is a critical need to improve our understanding of the disease process as well as the appropriate prevention and treatment.

The purpose of this prospective study was to identify the CPSP incidence in elderly patients at both 3 and 6 months after operation. We also intended to clarify the neuropathic component of the pain, to assess its interference on individual activities of daily living (ADL), and to determine the risk factors of chronicity of postoperative pain.

## Methods

### Participants

This study was approved by the hospital Ethics Committee of The First Affiliated Hospital of Chongqing Medical University, China. Written informed consent was obtained from all of the participants. Patients aged ≥ 60 years with American Society of Anesthesiologists (ASA) status between I and III, ability to understand the information sheet, undergoing elective surgery in our institution between April 2018 and March 2020 were enrolled in this study. Patients with previous diagnosis of stroke and/or dementia, day case surgery, cardiac or intracranial surgery, or inability to complete a personal or telephone interview, major uncorrected vision or hearing loss, or major psychiatric disease were excluded. An appropriately trained clinical research assistant (CRA) who did not participate in perioperative management of the patients assisted the main investigators with subject enrollment and follow-ups.

### Preoperative assessment

After admission, the CRA made a face-to face interview with each participant. The questionnaire included items regarding age, sex, weight, height, living situation, preoperative pain at the site of later surgery or elsewhere, current smoking status, and pre-surgical comorbidities including diabetes, hypertension, cardiovascular disease and osteoarthritis.

Psychological well-being of the participants was assessed by with Chinese version of Hospital Anxiety and Depression Scale (HADS) [21]. It is a self-administered instrument to examine features of anxiety (7 questions) and depression (7 questions). Each subscale is a 4-point Likert scale scored from 0 to 3 [22]. According to the results of previous research, clinically relevant anxiety/depression was diagnosis with the threshold of ≥ 8 points in each subscale [23]. The Chinese version of HADS showed adequate validity in previous studies with favorable sensitivity and specificity for screening for psychological disorders [21, 24]. Furthermore, the scale was demonstrated to possess good reliability with a Cronbach’s alpha of 0.88 and a test–retest correlation coefficient of 0.94 [25].

### Surgery and anesthetic management

On the surgical day, data on the anesthetic (general anesthesia or regional anesthesia) and surgical procedures (type and duration) were collected. On postoperative day 1and 2, acute pain during both rest and movement were evaluated with numerical rating scale (NRS) ranged between 0 and 10 (0 indicates no pain, 10 indicates worst pain) every 12 h. The average pain severity within postoperative 24 h was calculated.

### Chronic pain measurement

The same blinded CRA conducted telephone follow-up interviews at 3 and 6 postoperative months. Patients were inquired whether they had any pain in surgical area and if the pain developed postoperatively. If subjects answered with a ‘no’ to any of the two questions, those patients were classified as cases without CPSP. Contrarily, if subjects answered yes, they were considered CPSP cases. For those patients reporting pain, additional measures were used, which focused on the assessment of CPSP characteristics.

We adopted the Chinese version of Brief Pain Inventory-Short Form (BPI-SF) to assess the features of CPSP [26]. BPI-SF is an instrument measuring the characteristics of postoperative pain in adults. It includes 4 questions on pain features, 7 questions on pain interference with ADL [27]. The Chinese version of BPI-SF was found to have good internal consistency with the intra-class correlation coefficient for the test–retest reliability of 0.79 for the pain severity scale and 0.81 for the pain interference scale [28].

The intensity of rest pain and movement pain during the past week of the interview was measured by NRS score. Pain intensity was divided into no pain (0), mild (1–3), moderate (4–6), and severe (7–10). Then, the subjects were inquired about pain frequency, location, and pain medication. The NRS score was employed to assess 7 aspects of pain interference with the ADL including general activity, mood, walking ability, normal work, relations with others, sleep, enjoyment of life (0 indicates no interference, 10 indicates complete interference).

In addition, patients with CPSP also completed the Chinese version of Douleur Neuropathique 4 questions (DN4) questionnaire to evaluate neuropathic features of the pain. The DN4 questionnaire is an instrument to evaluate the neuropathic component of chronic pain in adults. It comprises 3 items regarding category of pain including burning pain, cold pain, electric shock pain; 4 items regarding accompanied manifestations including tingling, pins/needles, numbness, itching; 2 items regarding the co-existence of numbness on contact or pinching, and 1 item regarding the initiation or aggravation of pain on rubbing [29]. Items from 1 to 7 of the DN4 questionnaire were answered with yes or no by interviewing patients. Items from 8 to 10 require physical examination performed by a professional person, but for the uses of the present study, it was adapted to complete by the patient. The patients were asked if their pain area was sensitive to touch, sensitive to pin prick, and sensitive to light brushing, similar to a clinical examination. Neuropathic pain was diagnosed as the pain of any intensity with a score ≥ 4 on the DN4 questionnaire. The Chinese version of the DN4 was proved to be a valid and reliable tool for diagnosing neuropathic pain with a Cronbach’s alpha of 0.85, and a sensitivity of 77% and specificity of 78% [30].

### Outcome variables

The primary outcome of this study was the incidence of CPSP at 3 postoperative months. CPSP was defined as the pain developing after surgery in or near the surgical location and last for ≥ 3 months (NRS score of > 0).

Secondary outcomes were the incidence of CPSP at 6 postoperative months, pain intensity, pain interference with ADL, and incidence of neuropathic pain at 3 and 6 months.

### Statistical analysis

The sample size was calculated according to the assumption that at least 10 cases were required to test every potential independent variable included in a multivariate logistic regression [31]. Based on the results of previous literature, the estimated incidence of CPSP at 3 months in older patients is around 30% [17, 19]. With a sample size of 800 cases (30% incidence), 240 cases were estimated to suffer from CPSP in this study. As a result, 24 potential factors can be enrolled in the multivariate logistic regression analysis with such a number of events.

Continuous variables were presented as mean ± standard deviation (SD). Categorical variables were shown as numbers (percentages). Between CPSP and non-CPSP groups, continuous variables were compared using independent samples *t*-test, and categorical variables were compared using Chi-squared test. After univariable analysis, variables were taken into multivariate logistic regression when *P* < 0.20 to determine the independent risk factors for chronic pain. Odds ratio (OR) and its 95% confidence interval (95% CI) were calculated to identify the impact of each variable on the development of CPSP. SPSS v.18.0 (IBM, Armonk, New York, United States) was used for data analysis. A *P*-Value of < 0.05 was considered as statistical significance.

## Results

### Baseline Characteristics

**Fig. 1:**
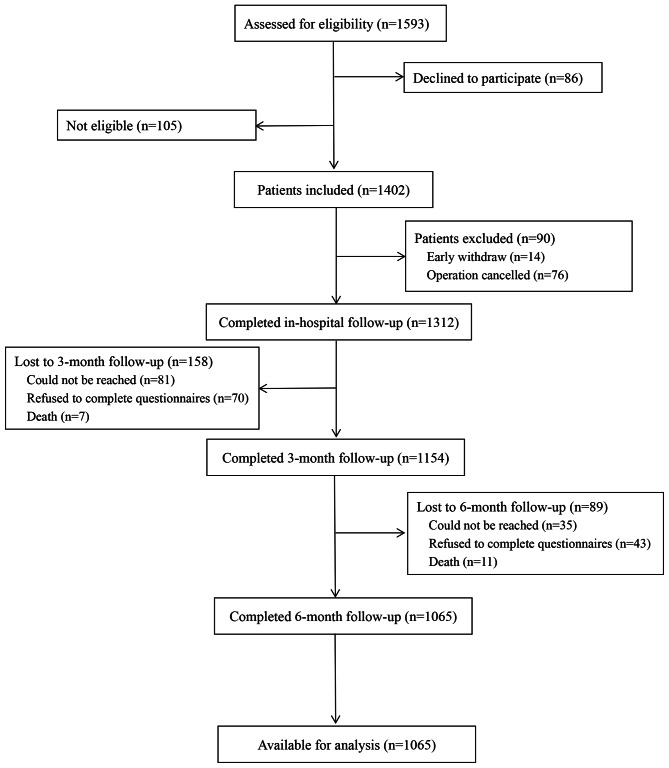
Flowchart of participants throughout the study

During the 24-month study period, 1593 patients were assessed for eligibility, of which, 86 patients declined participation and 105 patients were not eligible for this study. The main causes of ineligibility were discharge on the surgical day, cognitive/hearing/vision deficit, or inability to understand the study. Of the 1402 patients, 90 dropped out during inpatient follow-up, 158 and 89 cases lost follow-up after 3 and 6 months, respectively. As a result, 1065 patients completed the whole follow-up period (Fig. 1). The baseline characteristics of the subjects and data on CPSP incidence are presented in Table 1. The mean (SD) age at the time of enrollment was 70 years, and the majority of the elderly patients were females (53.8%). The two main types of surgery were general procedure (33.1%) and orthopedic procedure (31.8%).

**Table 1:** Baseline demographic, clinical characteristics and CPSP prevalence (n = 1065)

Item	Value
Age (year, mean ± SD)	70 ± 8
Sex, n (%)	
Male	492 (46.2)
Female	573 (53.8)
BMI (Kg/m^2^, mean ± SD)	24.0 ± 2.2
Living alone, n (%)	
No	778 (73.1)
Yes	287 (26.9)
Preoperative pain at surgical site, n (%)	
No	610 (57.3)
Yes	455 (42.7)
Preoperative pain elsewhere, n (%)	
No	793 (74.5)
Yes	272 (25.5)
Current smoker, n (%)	
No	856 (80.4)
Yes	209 (19.6)
Diabetes, n (%)	
No	724 (68.0)
Yes	341 (32.0)
Hypertension, n (%)	
No	577 (54.2)
Yes	488 (45.8)
Coronary heart disease, n (%)	
No	826 (77.6)
Yes	239 (22.4)
Osteoarthritis, n (%)	
No	931 (87.4)
Yes	134 (12.6)
ASA physical status, n (%)	
II	488 (45.8)
III	549 (51.5)
IV	28 (2.6)
HADS-A score, n (%)	
< 8	722 (67.8)
≥ 8	343 (32.2)
HADS-D score, n (%)	
< 8	671 (63.0)
≥ 8	394 (37.0)
Type of surgery, n (%)	
Urological	155 (14.6)
Breast	60 (5.6)
Thoracic	158 (14.8)
General	353 (33.1)
Orthopedic	339 (31.8)
Surgical approach, n (%)	
Minimal invasive	652 (61.2)
Open	413 (38.8)
Type of anesthesia, n (%)	
General	959 (90.0)
Regional	106 (10.0)
Nerve block use, n (%)	
No	591 (55.5)
Yes	474 (44.5)
Duration of surgery (min, mean ± SD)	125 ± 47
Average pain intensity at rest within 24 h postoperatively (mean ± SD)	2.0 ± 0.9
Average pain intensity on movement within 24 h postoperatively (mean ± SD)	3.9 ± 1.3
Surgical site infection, n (%)	
No	969 (91.0)
Yes	96 (9.0)
Pain at 3 months, n (%, 95%CI)	379 (35.6, 32.7–38.8)
Pain at 6 months, n (%, 95%CI)	229 (21.5, 19.0–23.9 )

### Incidence and Characteristics of CPSP at three and six months

At 3- and 6-months following surgery, 379 patients (35.6%, 95% CI 32.7–38.8%) and 229 patients (21.5%, 95% CI 19.0–23.9%) reported having chronic pain, respectively. Following urological, breast, thoracic, general, and orthopedic surgery, the incidences of CPSP were 27.7% (43/155), 38.3% (23/60), 34.8% (55/158), 26.6% (94/353), 48.4% (164/339) at 3 months, and then decreased to 14.8% (23/155), 25.0% (15/60), 20.9% (33/159), 14.2% (50/353), 31.9% (108/339) at 6 months, respectively. Data on pain intensity, frequency, analgesic consumption, pain interference with ADL, and as well as incidence of neuropathic pain in the individuals with CPSP are shown in Table 2. At 3 and 6 months postoperatively, majority of the patients suffering from CPSP reported mild rest pain; the percentages of moderate-to-severe pain on movement were 71.2% and 59.8%, respectively. Three months following surgery, the rate of CPSP patients taking pain medication was 42.2%, and the decreased to 23.6% at 6 months. Three months after surgery, majority of the CPSP patients experienced pain daily or several times per week. Six months following operation, near 2/3 of the CPSP patients complained that the pain occurred once to several times a week.

**Table 2:** Characteristics of CPSP at three and six months after surgery

	Pain at 3 months (n = 379)	Pain at 6 months (n = 229)
Pain intensity		
Pain at rest in past week		
NRS score (mean ± SD)	2.2 ± 1.3	1.7 ± 1.1
No pain	30 (7.9)	36 (15.7)
Mild pain (n, %)	274 (72.3)	175 (76.4)
Moderate pain (n, %)	75 (19.8)	18 (7.9)
Severe pain (n, %)	0	0
Pain during movement in past week		
NRS score (mean ± SD)	4.7 ± 1.3	4.2 ± 1.2
Mild pain (n, %)	72 (19.0)	80 (34.9)
Moderate pain (n, %)	270 (71.2)	137 (59.8)
Severe pain (n, %)	37 (9.8)	12 (5.2)
**Analgesic administration (n, %)**	160 (42.2)	54 (23.6)
**Pain frequency (n, %)**		
Constantly	86 (22.7)	25 (10.9)
Daily	124 (32.7)	37 (16.2)
Several times/week	99 (26.1)	91 (39.7)
Once a week	57 (15.0)	56 (24.5)
Less than once a week	13 (3.4)	20 (8.7)
**Pain interference with activities of daily living**		
General activity	109 (28.8)	54 (23.6)
Mood	151 (39.8)	68 (29.7)
Walking ability	122 (32.2)	60 (26.2)
Normal work	78 (20.6)	37 (15.3)
Relations with other people	94 (24.8)	46 (20.1)
Sleep	92 (24.3)	58 (25.3)
Enjoyment of life	107 (28.2)	52 (22.7)
**Neuropathic pain (n, %)**	171 (45.1)	71 (31.0)

At postoperative 3 and 6 months, chronic pain significantly interfered with ADL of the patients. The patient’s mood was affected by the pain most frequently. Approximately 40% and 30% of the patients with CPSP reported that their mood was influenced by the pain at 3 and 6 months, respectively. CPSP seemed to have the least influence on normal work of the patients. Only about 20% and 15% of them complained that the pain influenced their normal work at 3 and 6 postoperative months (Table 2).

Table 2 presents that neuropathic pain (DN4 ≥ 4) was found in 171 of the 379 (45.1%) elderly patients with CPSP at 3 months. At 6 months, 31.0% of those with CPSP reported that the pain had neuropathic characteristics.

### Risk factors for chronic pain

Table 3 shows the univariate analyses results of potential risk factors for CPSP. Living alone, preoperative pain at surgical site or pain elsewhere, current smoker, history of osteoarthritis, preoperative anxiety, preoperative depression, surgical type, surgical approach, type of anesthesia, and higher pain severity on movement within postoperative 24 h were shown to be probable risk factors of CPSP at 3 or 6 months after surgery (*P* < 0.20).

**Table 3:** Univariate analyses of potential risk factors of CPSP at three and six months after surgery

	3 months			6 months		
	Chronic pain (n = 379)	No chronic pain (n = 686)	*P*-value	Chronic pain (n = 229)	No chronic pain (n = 836)	*P*-value
Age (year, mean ± SD)	70 ± 7	71 ± 8	0.336	70 ± 7	70 ± 8	0.379
Sex, n (%)			0.203			0.857
Male	185 (48.8)	307 (44.8)		107 (46.7)	385 (46.1)	
Female	194 (51.2)	379 (55.2)		122 (53.3)	451 (53.9)	
BMI (Kg/m^2^, mean ± SD)	24.1 ± 2.2	24.0 ± 2.1	0.308	24.1 ± 2.2	24.0 ± 2.1	0.377
Living alone, n (%)			0.155			0.290
No	267 (70.4)	511 (74.5)		161 (70.3)	617 (73.8)	
Yes	112 (29.6)	175 (25.5)		68 (29.7)	219 (26.2)	
Preoperative pain at surgical site, n (%)			< 0.001			< 0.001
No	180 (47.5)	430 (62.7)		103 (45.0)	507 (60.6)	
Yes	199 (52.5)	256 (37.3)		126 (55.0)	329 (39.4)	
Preoperative pain elsewhere, n (%)			0.229			0.104
No	274 (72.3)	519 (75.7)		161 (70.3)	632 (75.6)	
Yes	105 (27.7)	167 (24.3)		68 (29.7)	204 (24.4)	
Current smoker, n (%)			0.121			0.566
No	295 (77.8)	561 (81.8)		181 (79.0)	675 (80.7)	
Yes	84 (22.2)	125 (18.2)		48 (21.0)	161 (19.3)	
Diabetes, n (%)			0.438			0.455
No	252 (66.5)	472 (68.8)		151 (65.9)	573 (68.5)	
Yes	127 (33.5)	214 (31.2)		78 (34.1)	263 (31.5)	
Hypertension, n (%)			0.467			0.646
No	211 (55.7)	366 (53.4)		121 (52.8)	456 (54.5)	
Yes	168 (44.3)	320 (46.6)		108 (47.2)	380 (45.5)	
Coronary heart disease, n (%)			0.362			0.774
No	288 (76.0)	538 (78.4)		176 (76.9)	650 (77.8)	
Yes	91 (24.0)	148 (21.6)		53 (23.1)	186 (22.2)	
Osteoarthritis, n (%)			0.029			0.006
No	320 (84.4)	611 (89.1)		188 (82.1)	743 (88.9)	
Yes	59 (15.6)	75 (10.9)		41 (17.9)	93 (11.1)	
ASA physical status, n (%)			0.272			0.877
I	172 (45.4)	316 (46.1)		106 (46.3)	382 (45.7)	
II	193 (50.9)	356 (51.9)		116 (50.7)	433 (51.8)	
IV	14 (3.7)	14 (2.0)		7 (3.1)	21 (2.5)	
HADS-A score, n (%)			< 0.001			< 0.001
< 8	210 (55.4)	512 (74.6)		117 (51.1)	605 (72.4)	
≥ 8	169 (44.6)	174 (25.4)		112 (48.9)	231 (27.6)	
HADS-D score, n (%)			< 0.001			0.001
< 8	207 (54.6)	464 (67.6)		123 (53.7)	548 (65.6)	
≥ 8	172 (45.4)	222 (32.4)		106 (46.3)	288 (34.4)	
Type of surgery, n (%)			< 0.001			< 0.001
Urological	43 (11.3)	112 (16.3)		23 (10.0)	132 (15.8)	
Breast	23 (6.1)	37 (5.4)		15 (6.6)	45 (5.4)	
Thoracic	55 (14.5)	103 (15.0)		33 (14.4)	125 (15.0)	
General	94 (24.8)	259 (37.8)		50 (21.8)	303 (36.2)	
Orthopedic	164 (43.3)	175 (25.5)		108 (47.2)	231 (27.6)	
Surgical approach, n (%)			< 0.001			< 0.001
Minimal invasive	196 (51.7)	456 (66.5)		108 (47.2)	544 (65.1)	
Open	183 (48.3)	230 (33.5)		121 (52.8)	292 (34.9)	
Type of anesthesia, n (%)			0.877			0.149
General	342 (90.2)	617 (89.9)		212 (92.6)	747 (89.4)	
Regional	37 (9.8)	69 (10.1)		17 (7.4)	89 (10.6)	
Nerve block use, n (%)			0.230			0.288
No	201 (53.0)	390 (56.9)		120 (52.4)	471 (56.3)	
Yes	178 (47.0)	296 (43.1)		109 (47.6)	365 (43.7)	
Duration of surgery (min, mean ± SD)	123 ± 45	126 ± 45	0.335	127 ± 54	124 ± 45	0.416
Average pain intensity at rest within 24 h postoperatively (mean ± SD)	2.0 ± 0.9	2.0 ± 0.9	0.627	2.1 ± 0.9	2.0 ± 0.9	0.223
Average pain intensity on movement within 24 h postoperatively (mean ± SD)	4.2 ± 1.3	3.7 ± 1.3	< 0.001	4.3 ± 1.4	3.8 ± 1.3	< 0.001
Surgical site infection, n (%)			0.852			0.539
No	344 (90.8)	625 (91.1)		206 (90.0)	763 (91.3)	
Yes	35 (9.2)	61 (8.9)		23 (10.0)	73 (8.7)	

The multiple regression analysis indicated that preoperative anxiety, preoperative depression, orthopedic surgery, higher pain severity on movement within postoperative 24 h were associated with a higher risk for CPSP independently at both 3 and 6 months after surgery. In addition, general anesthesia was one of the risk factors for CPSP at 6-months postoperatively (Table 4).

**Table 4:** Multivariate logistic regression analysis of risk factors of CPSP at three and six months

		Pain at 3 months (n = 379)			Pain at 6 months (n = 229)	
		OR (95% CI)	*P*-value		OR (95% CI)	*P*-value
Living alone	No	1	0.228			
	Yes	0.829 (0.612–1.124)				
Preoperative pain at surgical site	No	1	0.444		1	0.813
	Yes	1.154 (0.800-1.665)			1.057 (0.666–1.679)	
Preoperative pain elsewhere	No				1	0.171
	Yes				1.289 (0.896–1.853)	
Current smoker	No	1	0.069			
	Yes	1.368 (0.976–1.918)				
Osteoarthritis	No	1	0.666		1	0.687
	Yes	1.094 (0.727–1.648)			1.099 (0.695–1.738)	
HADS-A score	< 8	1	< 0.001		1	< 0.001
	≥ 8	2.244 (1.693–2.973)			2.397 (1.745–3.294)	
HADS-D score	< 8	1	< 0.001		1	0.006
	≥ 8	1.709 (1.292–2.261)			1.565 (1.136–2.156)	
Type of surgery	Urological	1			1	
	Breast	1.400 (0.679–2.888)	0.362		1.510 (0.649–3.513)	0.339
	Thoracic	1.193 (0.721–1.973)	0.492		1.272 (0.693–2.336)	0.437
	General	0.754 (0.482–1.180)	0.217		0.766 (0.439–1.338)	0.349
	Orthopedic	1.927 (1.112–3.341)	0.019		2.484 (1.220–5.061)	0.012
Surgical approach	Minimal invasive	1	0.494		1	0.628
	Open	1.135 (0.790–1.630)			1.116 (0.715–1.744)	
Type of anesthesia	General				1	0.038
	Regional				0.515 (0.275–0.964)	
Average pain intensity at rest within 24 h postoperatively		1.317 (1.191–1.457)	< 0.001		1.317 (1.177–1.475)	< 0.001

## Discussion

In this prospective observational study, we found that CPSP is an important issue in elderly surgical patients, occurring in more than one third of patients (35.6%) 3 months following surgery. Six months later, 21.5% still complained of pain. Our study confirms that CPSP is common in elderly patients with a comparable incidence reported in previous studies. A multi-center retrospective study reported an incidence of 27.2% in patients aged > 65 years 6 months after hip arthroplasty [18]. A recent retrospective study, authors found that CPSP occurred in 30.8% of the elderly patients (aged ≥ 60 years), 3 months following orthopedic surgery [19]. In a community-based study, the rate of CPSP in elderly patients was 42% at approximately 3 months following hip fracture repair [16]. A prospective study that included 116 patients (aged ≥ 65 years) receiving major non-cardiac surgery reported that the incidence of CPSP was 42.5% at 3 months following surgery [17]. The variability in CPSP incidence across the studies originated from differences in study design, timing of pain evaluation, and definitions of chronic pain. In our study, only patients who completed follow-up interviews at both 3 and 6 postoperative months were included in data analysis. Although accuracy of the incidence of CPSP at 3 months might be potentially influenced, evolution of CPSP could be presented as a continuum. We found that the incidence of CPSP at 6 months was lower than that at 3 months following surgery. It could be speculated that spontaneous remission of chronic pain is natural because a tendency towards a reduction in CPSP over time was documented in previous long-term follow-up studies besides our findings [7, 32].

We found that majority of the CPSP patients experienced mild rest pain, and no patients underwent severe rest pain. Moderate-to-severe movement pain was common at 3 months after surgery, and then decreased obviously at 6 months. The intensity of the chronic pain in our study was comparable to other research [16]. However, comparison between literatures should be conducted cautiously due to the adoption of different pain assessment tools.

Chronic pain in the elderly could result in greater postoperative functional disability and poorer quality of life. Herrick et al. showed that moderate-to-severe pain was associated with difficulty in ADL 3 months after hip fracture repair in elderly patients [16]. Our study shows that the elderly patients who described pain had more restrictions in daily life activities as compared to the individuals who described no chronic pain. Negative impacts of CPSP on ADL were apparent in mood in patients suffered from CPSP. This exemplifies the consequences of chronic pain in mental well-being of this population.

The CPSP development is believe to be relevant to neuropathic mechanism involving nerves damage during surgery, and as well as neuroplastic changes after injury [33–35]. Redistribution of the voltage-gated sodium channel subtype in nociceptors expressing neuropathic physiology and microglial activation in the ipsilateral dorsal horn of the spinal cord occur at the cellular and tissue levels following nerve injury. These changes facilitate the development of persistent pain as they provoke long-term changes including altered gene expression in the dorsal root ganglia and the spinal cord, central sensitization, and trans-synaptic neurodegeneration [36, 37]. Few studies investigated the epidemiology of neuropathic pain following surgery in elderly individuals. In this study, the rate of CPSP with neuropathic component was lower than those reported in studies in young adult and pediatric patients [38, 39], although direct comparisons are not possible because of difference in patient characteristics. The protective impact of aging on the occurrence of neuropathic pain has been indicated in animal experiment, and the possible mechanisms include thickness of nerve sheath, nerve injury, and weaker glial activation [40].

Multiple perioperative risk factors of CPSP have been identified in non-age-stratified cohort studies, including biological factors (female sex, younger age), medical factors (greater preoperative pain, greater acute postoperative pain), and psychological factors (higher levels of pre-surgical anxiety and depression, pain catastrophizing) [2, 33]. In this study, we demonstrated that higher pre-surgical anxiety and depression, orthopedic surgery, and greater acute postoperative pain during movement were risk factors for CPSP in the elderly patients.

It has been widely studied that negative moods including anxiety and depression are relevant to reduced impacts on descending pain-inhibitory systems, enhancing spinal nociception and pain [41, 42]. The role of negative emotions in modulation of pain has been shown to be associated with impaired endogenous inhibition of pain coupled with central sensitization [43, 44]. Psychological interventions have been proved that influence supra-spinal mechanisms involving higher centers of pain, impacting endogenous pain modulation, thereby facilitating endogenous analgesia [45, 46]. As evidence is lacking to support the efficacy of systemic agents for CPSP prevention, targeting these psychological risk factors might lead to adaptive and functional modifications in pain processing, and thus to prevent the development of CPSP.

In the present study, patients undergoing orthopedic surgery had a high risk for CPSP. CPSP was found in 48.4% and 31.4% of the patients at 3 and 6 months after orthopedic procedures, respectively. Orthopedic surgery is often conducted because of pain, and preoperative chronic pain may confound the result. In this study, the multivariate analysis results indicated that orthopedic surgery patients were nearly 2.5 times more likely to develop CPSP than urological surgery patients after eliminating the impacts of confounders like preoperative pain. Our results are comparable with those of Fletcher et al., who showed that patients underwent orthopedic surgery had an about 3.4 times higher risk of CPSP than those underwent visceral surgery [47].

Consistent with previous studies conducted in adult and pediatric patients [3, 18, 39], our results indicated that the acute postoperative pain intensity during movement was a risk factor for CPSP at both 3 and 6 postoperative months in older patients. The underlying pathogenesis of CPSP might be attributed to inflammatory cytokines imbalance developed during surgery stress, thereby facilitates the sensitization of peripheral nervous system (PNS) and central nervous system (CNS) [48]. As acute postoperative pain could sensitize PNS and CNS, aggressive management of acute pain following surgery has been proposed to prevent the sensitization and reduce the risk of CPSP [2, 33, 49].

In this study, patients undergoing general anesthesia had a higher risk for CPSP at 6 postoperative months than those undergoing regional anesthesia. Regional anesthesia can be used as a tool in the prevention of CPSP because it has been proved to reduce the excitability of neurons involved in peripheral nociceptive pathways [33, 50]. Several studies have shown the beneficial results of regional anesthesia techniques in preventing the development of CPSP after hysterectomy, cesarean section, and total knee arthroplasty [51–53] .

We did not find independent associations between age, sex, preoperative pain at surgical site or pain elsewhere and CPSP. Although younger age, female sex, and preoperative pain are demonstrated as risk factors for CPSP in several studies [18, 54, 55], results are not conclusive in elderly cases [56]. Further research is required to address this issue. On the other hand, severity and distribution of preoperative pain, and preoperative functional status have been identified to be associated with the development of CPSP [57–59]. We only collected data on the presence of pain at surgical site or elsewhere at baseline, and did not gather information on the severity, frequency of preoperative pain, and its interference with activities of daily living of the patients. Therefore, the association between the features of preoperative pain and the development of CPSP could not be extensively explored in the present study. This together with other factors related to preoperative pain needs further investigation in the future.

Our study has several limitations. First, this is a single-country and single-site study, which limits the generalization of conclusions and compromises its external validity. Second, the information on CPSP were collected according to self-reporting of the patients by telephone interview, which may not avoid misinterpretation completely. Nonetheless, the follow-up interviews after discharge were undertaken with full caution to guarantee sufficient understanding of the answers and adequate control over the potential misunderstanding about the development of CPSP. Furthermore, we did not confirm the neuropathic characteristics of the pain reported by the subjects with physical examinations or quantitative sensory experiments, which might underestimate patients with clinician-diagnosed neuropathic pain. Although the present study provides important information about neuropathic features of CPSP in elderly patients, knowledge must be improved by further research on the topic. As the accuracy of screening self-questionnaires for neuropathic pain is imperfect, more information is needed about the likelihood of development of neuropathic component of the pain. For this, large cohorts with a long follow-up research with a confirmation of the diagnosis by clinical examination or quantitative sensory experiments are mandatory. Third, although around one-third of the patients in this study had a history of diabetes, we did not collected data on neuropathic features of preoperative pain at baseline. Given the comorbidities between diabetes and neuropathic pain, this makes it more likely that pre-existing diabetic neuropathy confounds the rate of CPSP with neuropathic component. Therefore, the potential impact of previous history of neuropathy on the development neuropathic features of CPSP should be further explored. Lastly, this study described CPSP after several types of surgical procedures. This heterogeneity might limit statistical associations and hamper the identification of specific factors.

Despite these limitations, our study represented an important evaluation of CPSP in the elderly surgical population. Our findings suggest that great efforts should be taken to improve perioperative management to reduce the incidence of CPSP, especially after orthopedic procedures. In the future, it would be essential to create a multi-disciplinary geriatric pain treatment and prevention program for the elderly individuals at risk of CPSP.

## Conclusions

In summary, our study indicated that the incidence of CPSP in elderly patients was 35.6% at 3 months, and then decreased to 27.1% at 6 months. Signs of neuropathic pain were reported in 45.1% and 31.0% of the cases with CPSP at 3 and 6 postoperative months, respectively. Preoperative anxiety and depression, orthopedic surgery, and greater intensity of acute postoperative pain on movement are associated with an increased risk for CPSP. It should be kept in mind that developing psychological interventions to reduce anxiety and depression and optimizing the management of acute postoperative pain will be effective in reducing the development of CPSP in this population.

## Data Availability

The data used to support the findings of this study are included in the article. Further inquiries can be directed to the correspondence author.
